# Simaba Cedron in Intermittent Fever

**Published:** 1855-04

**Authors:** 


### ECLECTIC DEPARTMENT,

### AND SPIRIT OF THE MEDICAL PERIODICAL PRESS.

---

*Simaba Cedron in Intermittent Fever.*—Dr. S. S. Purple, through the pages of the New York Journal of Medicine, calls attention to the virtues of the above article, in intermittent fever. The seeds of the plant were proposed some years since, as an antidote for the poison of venomous serpents. It had also been employed successfully in Central America in cases of intermittent fever, some of which had resisted the action of quinia. Dr. Purple's experience with the remedy has been favorable, and his investigation into its medical history and effects leads him to say:

From the declared experience of various observers of the medicinal effects of the Simaba cedron, we are warranted in drawing the following conclusions regarding its therapeutical action:

That it possesses decided anti-periodic properties, and is, therefore, applicable in the treatment of periodic diseases.

That it is less likely than quinine to produce the aggregate of encephalic or neuropathic phenomena, induced by overdoses.

That it may, in large doses, repeated often, produce griping of the bowels, and even diarrhœa; but that these conditions are easily controlled by appropriate medicaments.

That, as a remedy in intermittent fever, it possesses properties, in many respects, equal to quinine, and in most cases is equally adapted to the curation of this disease.

That, in the treatment of yellow fever, it does not appear to possess any particular advantage over quinine, but, nevertheless, is equally well adapted to fulfill the indications which call for the use of this latter remedy.

That it possesses marked tonic properties, and deserves a prominent place in this classification of the Materia Medica.

That in chronic dysentery, diarrhœa, dyspepsia, and all states of the stomach accompanied with impaired or difficult digestion, its use will be found to be attended with benefit.

That, should a demand arise for its use in medicine, it is believed that it will be found not difficult to obtain a supply, in quantities sufficient to afford it at much less price than quinine.—*N. Y. Jour. of Medicine*, from *New Jersey Med. Reporter*.

